# Metaverse in Medical Education: A Paradigm Shift

**DOI:** 10.12669/pjms.40.1.8752

**Published:** 2024

**Authors:** Khadija Farrukh

**Affiliations:** 1 Dr. Khadija Farrukh, Head of Department, Department of Medical Education, Bahria University Health Sciences Campus, Karachi, Pakistan. Email: Khadijafarrukh2010@hotmail.com

The concept of the metaverse in medical education is an exciting and innovative idea that has the potential to revolutionize how medical professionals are trained and educated. The metaverse refers to a collective virtual shared space, created by the convergence of virtually enhanced physical and digital realities.^1^ In the context of medical education, the metaverse can offer several advantages. Medical students can immerse themselves in highly realistic and Interactive virtual environments that simulate clinical settings. This can include virtual hospitals, operating rooms, and patient encounters. Such environments can help students gain practical experience and confidence in a controlled, risk-free setting.^2^

The metaverse can facilitate collaborative learning experiences among medical students and professionals from around the world. They can interact in real-time, discuss cases, and share knowledge, which can enhance their understanding and problem-solving skills. In the metaverse, medical students can encounter rare medical cases and scenarios that they might not come across in traditional clinical settings.^3^ This exposure can help them develop a broader understanding of medical conditions and their treatments. The metaverse can enable remote learning, making medical education more accessible to individuals in remote or underserved areas. Students can participate in virtual lectures, discussions, and simulations without the need for physical presence. Healthcare professionals can use the metaverse for ongoing skill development to stay up-to-date with the latest medical procedures and technologies. Metaverse platforms can employ artificial Intelligence and machine learning algorithms to tailor educational content to individual learners. This can help students learn at their own pace and focus on areas where they need the most improvement.

Virtual simulations within the metaverse can be used for objective assessments of students’ clinical skills and decision-making abilities. This can provide valuable feedback to both students and educators. Medical students and professionals can build a global network of peers and mentors through metaverse-based communities and events, fostering international collaborations and knowledge exchange. The metaverse roadmap categorizes the metaverse into four types: augmented reality, lifelogging, mirror world, and virtual reality.

Osso VR is a VR-based platform that allows medical professionals, including surgeons, to practice and improve their surgical skills in a virtual environment. Holo anatomy is VR application provides medical students with an immersive way to study human anatomy by using Microsoft’s HoloLens mixed reality headset. Research is being conducted to assess the impact of metaverse-based interprofessional education, where medical students collaborate with professionals from other healthcare disciplines in virtual settings to improve teamwork and communication skills. Researchers are exploring how artificial intelligence-driven adaptive learning systems within the metaverse can personalize medical education content to individual learners, addressing their specific needs and areas of improvement. Research also delves into the ethical and privacy concerns associated with using metaverse platforms in medical education, including issues related to patient data and confidentiality. Research aims to measure the long-term impact of metaverse-based education on the clinical competency and performance of medical professionals. Challenges in implementing the metaverse in medical education include concerns about the cost of technology, ensuring the security and privacy of patient data in virtual environments, and the need for rigorous validation of virtual simulations to ensure they accurately reflect real-world medical scenarios.
